# Pressure Field Assisted Polycondensation Nonaqueous Precipitation Synthesis of Mullite Whiskers and Their Application as Epoxy Resin Reinforcement

**DOI:** 10.3390/polym11122007

**Published:** 2019-12-04

**Authors:** Guo Feng, Feng Jiang, Zi Hu, Weihui Jiang, Jianmin Liu, Quan Zhang, Qing Hu, Lifeng Miao, Qian Wu, Jian Liang

**Affiliations:** 1 National Engineering Research Center for Domestic & Builing Ceramics, Jingdezhen Ceramic Institute, Jingdezhen 333000, China; liujianmin@jci.edu.cn (J.L.); zhangquan@jci.edu.cn (Q.Z.); miaolifeng@jci.edu.cn (L.M.); wuqian@jci.edu.cn (Q.W.); liangjian@jci.edu.cn (J.L.); 2 Department of Material Science and Engineering, Jingdezhen Ceramic Institute, Jingdezhen 333000, China; huqing@jci.edu.cn; 3 Jiangxi Ceramic Research Institute, Jingdezhen 333000, China

**Keywords:** mullite, whiskers, nonaqueous precipitation method, aluminum fluoride, polar transformation, screw

## Abstract

Mullite whiskers were novelty prepared via pressure field assisted polycondensation nonaqueous precipitation method. The precipitate phase transition in heating process, phase compositions and microstructure of samples calcined at different temperatures, effect of pressure field on precursors polycondensation and AlF_3_ amount on sample morphology, the structure and the growth mechanism of whiskers were investigated. The results indicate that pressure field caused by kettle treatment promotes the polycondensation reaction between AlF_3_ and tetraethyl orthosilicate (TEOS), the excess aluminum fluoride coordinates with the precipitate skeleton of the =Al–O–Si≡, which brings about the low mullitization temperature (900 °C). The sample prepared with the optimal amount of aluminum fluoride (1.3 of the theoretical amount) calcined at 1100 °C presents high yield and aspect ratio (>15, 100 nm in diameter) of mullite whiskers. Growth of whiskers prepared via pressure field assisted polycondensation nonaqueous precipitation method is attributed to a vapor-solid (VS) mechanism with the inducement of screw. These mullite whiskers with the structure of multi-needle whiskers connected in the same center can be distributed evenly in epoxy resin, which greatly improves the mechanical properties of epoxy resin.

## 1. Introduction

Mullite (3Al_2_O_3_·2SiO_2_) materials are widely applied as high-temperature engineering and refractory materials, due to their unique excellent properties of high-temperature strength, low thermal conductivity, high creep resistance, relatively low thermal expansion coefficient, excellent chemical stability and creep resistance [1,2]. Mullite has a stable crystal structure of orthorhombic. Its lattice constants (a, b and c) are of 7.545 Å, 7.689 Å and 2.884 Å (JCPDS Card # 15-0776). The crystal growth of mullite is generally more quickly in the c-axis direction than any other direction, which brings a high orientation degree to form mullite whiskers. Mullite whiskers have attracted much attention as the reinforcement for high-temperature materials [3,4,5].

Various processing techniques have been utilized to prepare mullite whiskers, including the mineral decomposition method [6], vapor-phase reaction method [7], molten salt method [8], hydrolytic sol–gel method [9], nonhydrolytic sol–gel method [10], nonhydrolytic sol–gel combined with the molten salt method [11,12,13,14], etc. The nonaqueous precipitation method is a novel materials synthesis method, and it holds the merits of simple process, short cycle, non-aggregation of products [15,16,17]. However, in order to give full play to the advantages of nonaqueous precipitation method, the precursor materials must undergo nonhydrolytic polycondensation. In our previous researches, it was found that the polarity of precursor material should not be too large, the ion bond percentage should not be more than 50%, in order to make the precursor material directly undergo nonhydrolytic polycondensation. At the same time, these polar compounds with a large percentage of ionic bond are often characterized by low cost, low toxicity and environmental protection. However, it is a traditional problem in the field of nonhydrolytic polycondensation that how to make compounds with more than 50% ionic bond participate in nonhydrolytic polycondensation. If the compounds with high ionic bond percentage more than 50% can also participate in nonhydrolytic polycondensation, the range of raw materials for nonhydrolytic polycondensation can be greatly increased. In this work, a method of pressure field assisted polycondensationis developed to make the compound with more than 50% ionic bond percentage participate in nonhydrolytic polycondensation. This method is proposed to be used in mullite whiskers in-situ synthesis via a facile nonaqueous precipitation process with high ionic bond percentage. In comparison with traditional mullite whisker preparation methods, this in-situ nonaqueous precipitation method has the superiorities of low mullitization temperature, high homogeneity and efficiency, simple operation. The present work studies the phase transition process and the structure of precipitate. Effects of aluminum fluoride amount on whisker preparation are also investigated. The whiskers growth mechanism is discussed and their application as epoxy resin reinforcement is also investigated.

## 2. Materials and Methods

Analytical grade of anhydrous aluminum fluoride (AlF_3_), tetraethoxysilane (Si(OC_2_H_5_)_4_) and anhydrous ethanol (C_2_H_5_OH) were produced by China Medicine (Group) Shanghai Chemical Reagent, Co, Ltd. They were directly used without further purification.

In the glove-box, 13.5 mL tetraethyl orthosilicate (TEOS) was dissolved in 120 mL anhydrous ethanol with the formation of TEOS-ethanol solution (0.5 mol/L). Then 15.116 g (theoretical amount (TA) for mullite synthesis, A_0_), 16.628 g (1.1 of TA, A_1_), 18.139 g (1.2 of TA, A_2_), 19.651 g (1.3 of TA, A_3_) and 21.162 g (1.4 of TA, A_4_) anhydrous aluminum fluoride was added to the ethanol solution of TEOS, respectively. Mixtures were transformed to a kettle with the nominal volume of 200 mL, and then held at 130 °C for 12 h. After washed repeatedly with ethanol and filtered, the precipitate powders were obtained. They were finally dried at 110 °C for 2 h, and then calcined to a temperature scale from 900 to 1100 °C for 4h to get the final samples.

As to the epoxy resin samples, 5 g A_3_ mullite whisker (M^#^) or without whisker (E^#^), and 6 g diethanolamine were weighed and added to 5 0g epoxy resin with continuous and fierce stirring. After the mixture was stirred evenly, they were poured into tin paper mold. The samples were then degassed for 1 h in air and 30 min in negative pressure of 0.06 MPa, and cured at 80 °C for 12 h to get the final sample.

Crystal phases of the samples calcined at different temperatures were tested via XRD (X-ray diffractometer, D8, Bruker, Karlsruhe, Germany) with radiation of CuK*_α_* operated at 30 mA and 40 kV. The bonds contained in the precipitates were determined via FT-IR (Fourier transform infrared spectroscopy, Nicolet 5700, Thermo, Boston, Massachusetts, USA) in the wavenumber of 4000–400 cm^−1^. The samples morphology with different aluminum fluoride amounts was characterized by FE-SEM (field-emission scanning electron microscopy, SU-8010, JEOL, Tokyo, Japan). The whiskers structure was determined via TEM (transmission electron microscopy, JEM-2010, JEOL, Tokyo, Japan).

The mechanical properties of pure epoxy resin and mullite whisker-epoxy resin composite were measured by universal testing machine (TH-8203, Suzhou, China). Two-body abrasive wear test was determined by pin-on-disc machine (MPX-2000A, Zhangjiakou, China) under multi-load conditions. The specimens with the size of 10 mm × 10 mm × 3 mm and surface rubbed were glued on the steel sample clip. They were rubbed with the abrasive paper of SiC, which was pasted on the disc through the adhesive.

The specific abrasive wear rate (*W_S_*) was calculated by the following relation:

(1) $$W_{s}=K\frac{V_{S}V_{C}}{E\;H\varepsilon_{f}\mu_{\alpha }{\;F}_{N}},$$

where the *K* is proportionality constant, *H* is the hardness, *E* is the elastic modulus, *ε_f_* is the failure strain, *μ_α_* is the friction coefficient, *F_N_* is the normal load, *V_S_* is the sliding speed and *V_C_* is the crack growth speed. In present work, the *V_S_* (150 rpm) and the *F_N_* (5, 10 and 15 N) are small. It is applicable of Equation (1).

## 3. Results and Discussion

### 3.1. Precipitate Phase Transition Analysis

**Figure 1:**
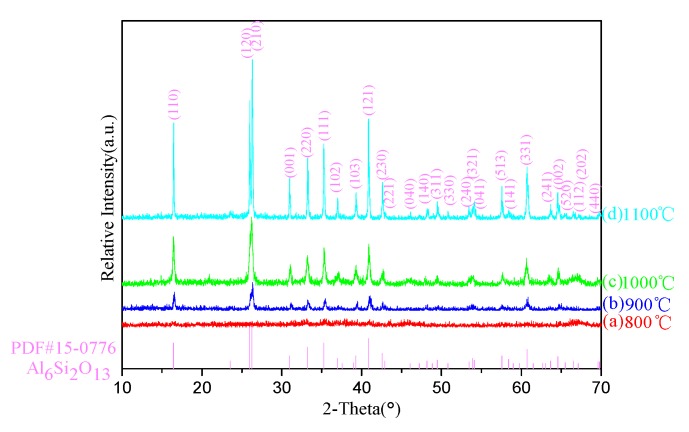
XRD patterns of the samples calcined at different temperatures.

Figure 1 presents the XRD patterns of A_3_ precipitate calcined in the temperature range of 800–1100 °C with intervals of 100 °C. As can be seen from Figure 1, the mullitization occurred at 900 °C, which could be inferred from the XRD pattern of the sample heated at 800 °C had no diffraction peak and sample heated at 900 °C presented a unique mullite phase without any other diffraction peak. It is worth noting the mullitization temperature is much lower than the traditional one [18] generally generated via the solid phase reaction between SiO_2_ and Al_2_O_3_ according to formula (2), which is benefited from the precipitate mullitization is generated by the =Al–O–Si≡ bonds rearrangement confirmed by the later FT-IR analysis.

3Al_2_O_3_ + 2SiO_2_→Al_6_Si_2_O_13_. (2)

As the temperature further increased to 1100 °C, it is also noteworthy that no impurity phase peak, such as Si-Al spinel or *γ*-Al_2_O_3_, could be observed in the XRD patterns from 900 to 1100 °C. Mullite is a unique phase appeared in the whole heating process. These indicate TEOS had completely reacted with AlF_3_, and the excessive AlF_3_wasto the point.

### 3.2. Effect of Pressure Field Caused by Kettle Treatment

**Figure 2:**
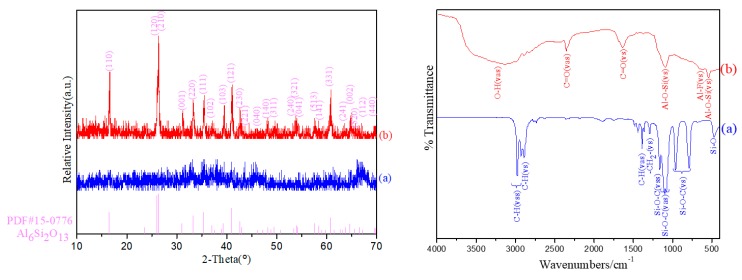
XRD patterns and FT-IR spectra of the samples without kettle treatment (**a**)and with kettle treatment (**b**).

Figure 2 shows the XRD patterns of the samples without kettle treatment (a) and with kettle treatment (b), both of them were calcined at 900 °C. No crystal phase diffraction peak existed in the sample prepared without kettle treatment (a), indicating that the sample was amorphous without the formation of mullite phase. In stark contrast, only mullite phase diffraction peaks were detected in the sample prepared with kettle treatment (b), suggesting that the crystalline phase of the sample was the pure mullite phase. Figure 2 also presents the FT-IR spectra of precipitates without kettle treatment (a) and with kettle treatment (b) to study chemical root for low mullitization temperature of the sample prepared with kettle treatment. The FT-IR spectrum of the sample without kettle treatment (a) shows a typical FT-IR spectrum of TEOS, the characteristic vibrations of Si–O–C in TEOS are shown in it. However, no aluminum related vibration was detected, indicating that AlF_3_didnot participate in reaction. It was mainly because the ionic character of Al–F bond calculated according to formula (3) was 64.89%. It indicates an obvious ionic character of Al–F bond. AlF_3_ preferred to exist in ions form in precursors mixtures liquid theoretically in conventional conditions. In sharp contrast, the FT-IR spectrum of precipitates with kettle treatment shown in Figure 2 presents typical absorbance peaks of =Al–O–Si≡. It is the intermediate product of reaction between AlF_3_ and TEOS. The vibrations at 492 cm^−1^ and 1044 cm^−1^ ascribed to δ(SiO_4_) and ν(SiO_4_) indicate the silica tetrahedron formation. This certificates that molecules TEOS had reacted with (partial of) AlF_3_ molecules completely. The vibrations at 810 and 854 cm^−1^ assigned to ν(AlO_4_) also show that the three Fs bonded with Al in partial of AlF_3_ molecules were completely replaced by the groups of Si–O. While the appearance of Al–F bond at 607 cm^−1^ was caused by the excess of AlF_3_.In addition, the C=O bonds in sample (b) were caused by adsorption of carbon dioxide in air, which indicates that the precipitate skeleton had higher coordination ability and polycondensation.

**Figure 3:**
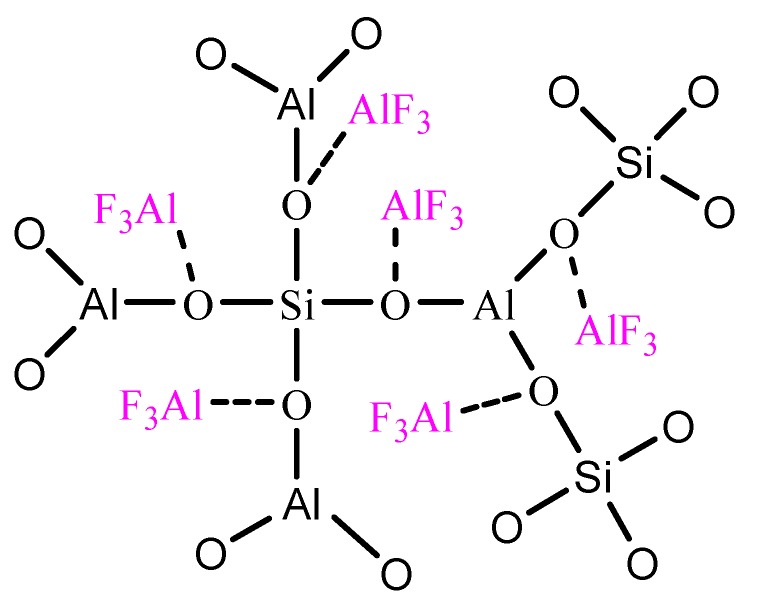
Deduced structure of precipitate with kettle treatment.

These results indicate the reaction between AlF_3_ and TEOS shown as formula (3) and the structure of precipitate with kettle treatment is deduced as shown in Figure 3.

Ionic character percentage (%) = 1 − exp[−(*X_A_* − *X_B_*)^2^/4]. (3)

In formula (3), *X_A_* and *X_B_* are the electronegativities of the two elements in the compound AB.

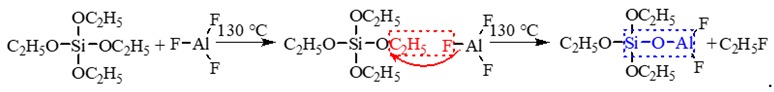
(4)

The polarity of the compound can be reversed in organic chemical synthesis reactions. The concept of “polar transformation” in the reaction has been paid attention to in recent years. It is generally believed that the change of entropy (isothermal) and temperature (adiabatic) will induce the change of dipole state when the molecular dipole of the material changes from a disordered state to an ordered state. If the temperature change and the entropy change are large, it is called the electrothermal effect of the material. Electric dipoles undergo heating fluctuations without external pressure, and their orientations are random, similar to those of water molecules. When molecules are subjected to external pressure, the dipoles may turn. When the external pressure becomes high enough, the dipole can even be completely symmetrical, and the material is polarize-saturated to form polar covalent bonds with high covalent bond percentage. Polarity conversion can broaden the selections of raw materials in the organic related synthesis process. Therefore, when designing the reaction process, it should consider not only the nature of the raw material itself and the inherent performance in the reaction but also the possible transformation of raw material in the reaction process.

“Polar transformation” can further enrich the contents of nonhydrolytic reactions and discussion on the polarity of raw materials from the perspective of environment temperature and pressure. It plays an important role in mastering the organic synthesis and provides a simpler way for the synthesis of new compounds. It also enriches raw materials selection for nonhydrolytic reactions.

### 3.3. Effect of AlF_3_Amount on Sample Morphology

**Figure 4:**
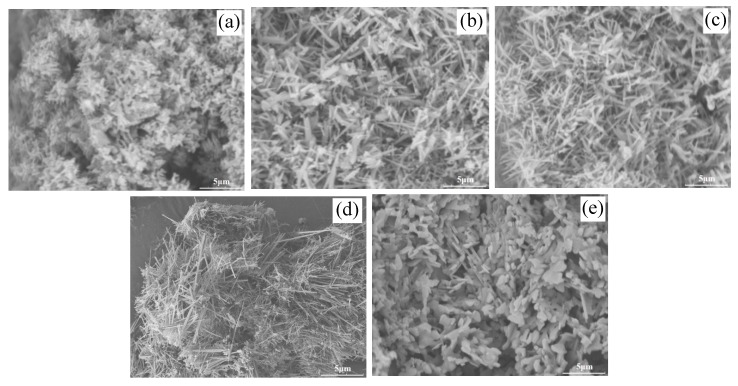
Field-emission (FE)-SEM images of the samples with different AlF_3_ amounts; (**a**) A_0_, (**b**) A_1_, (**c**) A_2_, (**d**) A_3_ and (**e**) A_4_.

Figure 4 presents the FE-SEM graphs of the samples prepared with different AlF_3_ amounts, A_0_ (a; 1.0 theoretical amount (TA)), A_1_ (b; 1.1TA), A_2_ (c; 1.2TA), A_3_ (d; 1.3TA) and A_4_ (e; 1.4TA). Figure 4a shows characteristic powder morphology; it is similar to the powder morphology prepared via a non-hydrolytic sol–gel method [19]. The sample presents rice-like grains with the one-dimensional growth trend. In comparison Figure 4a–d, the latter three samples show the characteristic whisker-like morphology. Figure 4b–d) show that the particles mingled gradually disappeared in whiskers with the increase of AlF_3_ amount. The whiskers diameter further increased with the AlF_3_ amount increase. The sample finally shows cluster-like-structured whisker morphology. However, when the amount of AlF_3_ further increased to 1.4TA (A_4_; e), mullite whisker had disappeared. Sample morphology develops into a sheet-like structure with a thickness of about 0.1–0.25 μm. When AlF_3_ was used as a vapor catalyst for crystal growth, vapor saturation was the decisive factor for the final products. Only appropriate low vapor saturation could generate high-quality whiskers. In this work, when AlF_3_ amount equaled to the theoretical amount for mullite synthesis, the whole system had no AlF_3_ vapor phase. Mullite crystal could hardly grow preferentially and finally formed a particulate. For sample A_1_ and sample A_2_, they were in low vapor concentration, their mullitization and mullite whiskers growth were limited. The limited mullitization and mullite whiskers growth led to the mullite whiskers being mixed with mullite particles. However, when the AlF_3_ amount further changed to 1.4TA, the vapor supersaturation concentration of AlF_3_wasat an over high degree, AlF_3_ preferentially reacted with H_2_O vapor to generate alumina phase. This process is shown in formula (5) and formula (6). Al_2_O_3_ preferred to grow into platelet-shaped corundum in AlF_3_ vapor. Consequently, the optimal AlF_3_ amount was 1.3TA, namely 1.3 of the theoretical amount for mullite synthesis.

AlF_3_ + H_2_O = AlOF + 2HF. (5)

2AlOF + H_2_O = Al_2_O_3_ + 2HF. (6)

### 3.4. Structure Analysis of Whiskers

**Figure 5:**
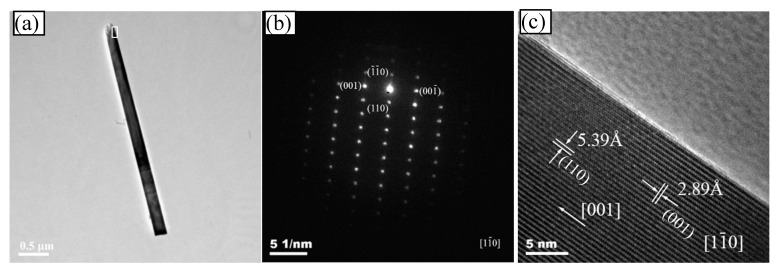
TEM, SAED and HR-TEM graphs of whisker. (**a**) TEM graph; (**b**) SAED pattern and (**c**) HR-TEM graph.

Figure 5 shows the TEM graph (a), SAED pattern (b) and HR-TEM graph (c) of the optimal A_3_ sample. TEM (a) graph shows the typical morphology of the as-prepared A_3_ mullite whisker. It demonstrated that the whisker had a relatively uniform microstructure, which shows the well-distributed whiskers had formed. The mullite whiskers were less than 100 nm in diameter and more than 15 in the aspect ratio. It was consistent with the results of FE-SEM. There was dark bands in the whisker center, which indicates that it was a solid whisker rather than a tube. SAED pattern (b) indicates the single mullite phase diffraction pattern. The whisker SAED pattern revealed a single diffraction pattern of the mullite phase. In the SAED pattern, the cell constants were measured a = 0.757 nm, b = 0.769 nm and c = 0.289 nm. They were in excellent agreement with the theoretical mullite values (JCPDF file no. 15-0776) and those calculated from XRD pattern. It was also deduced the SAED pattern that [1$\bar{1}$0] was the crystal band axis of mullite whiskers, and the axial diffraction spots along the whisker corresponded to [001]. The mullite whiskers growth direction was parallel to [001] direction. It was along the c axis direction. It was also proved by the HR-TEM graph shown in Figure 5c. The HR-TEM of sample also clearly revealed that the whisker was a perfect mullite monocrystal, without the crystal defects, such as low angle tilt grain-boundary or dislocation in the whisker.

### 3.5. Whiskers Growth Mechanism Analysis

**Figure 6:**
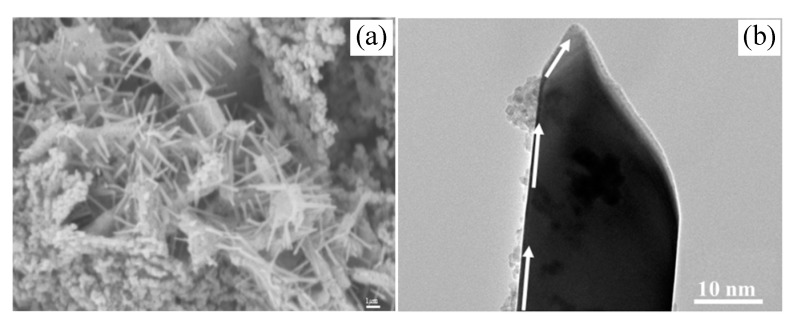
FE-SEM and TEM of the sample A_3_ holding at 1100 °C for 0.5 h; (**a**) FE-SEM and (**b**) TEM.

**Figure 7:**
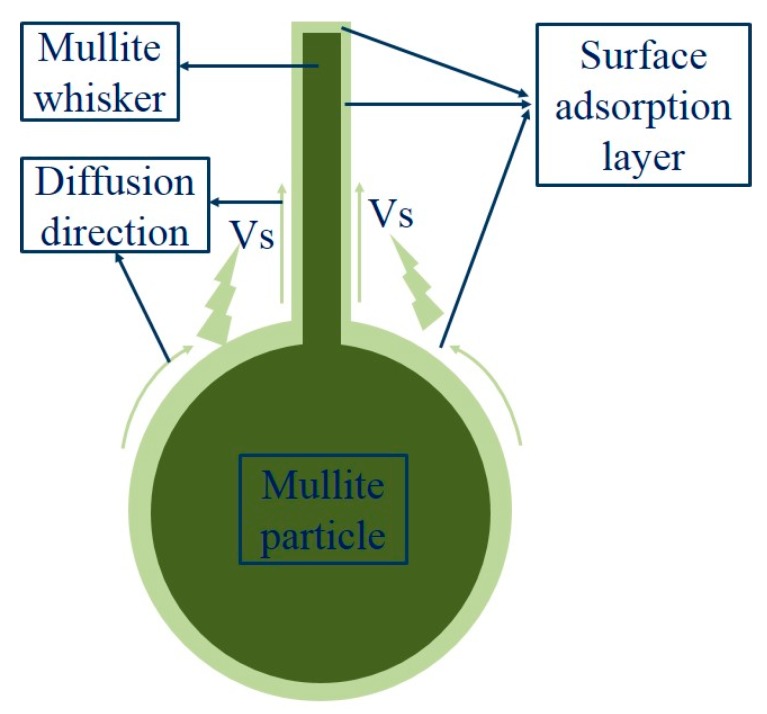
Schematic diagram illustrating whiskers growth mechanism.

To confirm the mullite whiskers growth mechanism prepared via nonaqueous precipitation in-situ synthesis method, Figure 6 shows FE-SEM (a) and TEM (b) graphs of the sample A_3_ held at 1100 °C for 0.5 h. Figure 6a presents many rod-like crystals on the gel skeleton surface. These crystals show an evident anisotropic growth trend. There are also many tiny dots on the particles. Figure 6b shows the high magnification TEM graph of the short-rod-like crystals shown in Figure 6a. A mass transport path appears in the direction shown by the white arrow in Figure 6b, combining with non-circular tip, which is generally thought to be relative to screw, these indicate the vapor–solid (VS) model with the inducement of screw for mullite whiskers prepared in this work. Based on the results and analysis above, a possible growth mechanism for mullite whiskers synthesized via nonaqueous precipitation method is schematically illustrated in Figure 7. Firstly, heterogeneous polycondensation reaction between the precursors of tetraethyl orthosilicate (TEOS) and aluminum fluoride occurred with the aid of kettle treatment. The heterogeneous polycondensation product =Al–O–Si≡ generated low-temperature nucleation of mullite, which was quite in favor to the further anisotropic growth of mullite. This low mullitization temperature also ensured enough three-dimensional growth dynamics differences, and it was also slightly lower than the volatilization temperature of aluminum fluoride. Secondly, it is shown in Figure 6b that slender whiskers had formed by calcining at 1100 °C for 0.5 h, which indicates vapor sediments on the surface of the whisker with sharp-pointed top, which indicates the formation of screw. These sediments with markedly surface diffusion sign were caused by the deposition of vapor molecule on the whisker surface. Figure 6b also shows the sediments diffusion sign and process along the whisker surface to the growth point. It is generally known that mullite whiskers growth is a dynamic physicochemical process, in which AlF_3_ is widely regarded as the most effective auxiliary for mullite whiskers growth [20]. The vapor phase diffusion is known as the main mass transport mechanism during the anisotropic growth of mullite whiskers, it can also induce the formation of screw, which is also beneficial to the mass transfer and whisker growth. The AlF_3_ vapor accelerates the mullite whiskers growth, because the mass transport for crystal growth is enhanced in the presence of vapor transport. The large mass transport accelerated by the vapor phase promotes the grains growth near the surfaces and the formation of whiskers (Figure 7). The growth structure at the end of whisker shown in Figure 6 was related to the enrichment of vapor nucleating particles during calcining process. At this time, the aggregates formed in the nucleating process could not crystallize along the orientation of whisker lattice, thus forming polycrystalline aggregation shown in Figure 6b. The further growth process could only occur on the small crystal surfaces with different orientations, that is, secondary growth at the end of the whisker. It is believed that the secondary growth phenomenon of mullite whisker may be due to the local wave of A1OF and SiF_4_ [2]. This is because when the top of the whisker enters a region with high vapor supersaturation, the vapor phase reactants with high supersaturation will nucleate rapidly at the top of the whisker (possibly with the help of many dislocation points at the top of the whisker), and form a polycrystalline pile-up pattern at the top of the whisker. With the formation of mullite crystal nucleus, the supersaturation of gas-phase reactants decreased rapidly and restored to the appropriate supersaturation for mullite whisker growth. The growth started from the different orientation of the microcrystalline surfaces in the polycrystalline aggregation surface, and it grew outwards continuously, forming the secondary growth of mullite whisker. There was avisible terminal-bottleneck phenomenon at the end of the whisker shown in Figure 6b. The terminal-bottleneck phenomenon detected in this work was generally thought as the most typical characteristic of vapor–solid (VS) whisker growth mechanism.

According to the results of this work, our previous researches [11,12,13,14,15] and reference [21], Figure 7 schematically illustrates the growth mechanism of mullite whiskers prepared by the nonaqueous precipitation method. Due to non-hydrolytic heterogeneous polycondensation reaction between ethyl silicate and aluminum fluoride shown in formula (3) with the help of kettle treatment, and the formation of =Al–O–Si≡ bonds in precipitate, the mullitization temperature is 900 °C. It was slightly lower than the volatilization temperature of aluminum fluoride. These could ensure when aluminum fluoride begins to volatilize, there are a large number of mullite crystal nucleus. Low mullitization temperature also ensures the appropriate three-dimensional growth dynamic differences. Meanwhile, residual aluminum fluoride in precipitate, which uniformly coordinates with the gel skeleton, volatilizes and reacts with O_2_ in the air according to formula (7) to form AlOF and F, and then =Al–O–Si≡ reacts with F to form AlOF and SiF_4_ [10,19]. They can further react with O_2_ to form mullite (shown in formula (8)). The newly formed mullite are generally on the surface of the particle, it plays the role of raw material for whisker growth and continuously diffuses to the top of the whisker with the help of gas-phase mass transport and concentration gradient. With the continuous reaction and mass transport process, precipitates particles eventually grow into mullite whiskers.

(7) $$=\mathrm{Al}–O–\mathrm{Si}\equiv \;(\mathrm{amorphous})\;+\;5F\to \mathrm{AlOF}+{\mathrm{SiF}}_{4}$$

(8) $$6\mathrm{AlOF}\;+\;2{\mathrm{SiF}}_{4}+\;3.5O_{2}\to 3{\mathrm{Al}}_{2}O_{3}\cdot 2{\mathrm{SiO}}_{2}(\mathrm{whisker})+14F.$$

### 3.6. Application of Whiskers in Epoxy Resin Reinforcement

**Figure 8:**
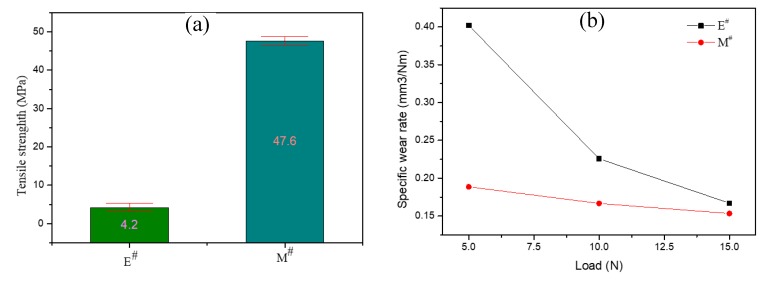
Mechanical properties of the samples. (**a**) The flexural strength of epoxy resin without mullite whisker (E^#^) and mullite whisker-epoxy resin composite (M^#^); (**b**) Specific abrasive wear rates of pure epoxy resin and mullite whisker-epoxy resin composite as a function of normal load.

Figure 8a shows the flexural strength of epoxy resin without mullite whisker (E^#^) and mullite whisker-epoxy resin composite (M^#^). For the epoxy resin, its flexural strength was 4.2 MPa (Figure 8 E^#^). The flexural strength for mullite whisker-epoxy resin composite (M^#^) dramatically increased to 47.6 MPa, which was 11.3 times of pure epoxy resin. The mullite whisker-epoxy resin composite (M^#^) also shows a lower relative flexural strength deviation, which might be due to the addition of mullite whiskers reduces the influence of defects on the materials. Specific abrasive wear rates of pure epoxy resin and mullite whisker-epoxy resin composite as a function of normal load are shown in Figure 8b. The abrasive wear rate of mullite whisker-epoxy resin composite was much smaller than that of pure epoxy resin. Both of their abrasive wear rates have presented a sharp decreasing trend with the load increasing, which is in excellent agreement with Lhymn’s mathematical model [22]. It was also obvious that effects of load on epoxy resin without mullite whisker (E^#^) were much larger than that of mullite whisker-epoxy resin composite (M^#^), which shows a strong reinforcement of mullite whiskers to epoxy resin.

**Figure 9:**
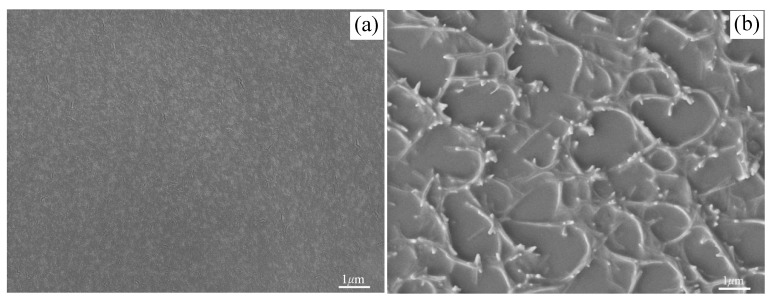
Of samples, (**a**) original epoxy resin and (**b**) mullite whisker-epoxy resin composite.

To confirm the reinforcement mechanism of mullite whisker to epoxy resin, the morphology of mullite whisker-epoxy resin composite is presented in Figure 9. Compared with the original epoxy resin shown in Figure 9a, the morphology of mullite whisker-epoxy resin composite clearly indicates that mullite whiskers were three-dimensionally distributed in epoxy resin. The multi-needle whiskers were connected in the same center, and there were whiskers in all directions, which could play a better synergistic role. They could prevent the generation and development of cracks and resist the damage of epoxy resin caused by friction. The unique structure of the multi-needle whiskers connected to the same center guarantees that when a whisker is exposed to shear and pressure, other whiskers will disperse the force of the whisker, thus eliminating stress concentration, preventing cracks and reducing the probability of damage.

## 4. Conclusions

Well-developed mullite whiskers were prepared via nonaqueous precipitation in-situ synthesis method at 1100 °C taking tetraethoxysilane (TEOS) as silicon source, anhydrous AlF_3_ as aluminum source and growth auxiliary for whisker. The precipitate was composed of =Al–O–Si≡ bonds and coordinated excessive AlF_3_. Kettle treatment facilitated the formation of =Al–O–Si≡ bonds due to it changing the polarity of aluminum fluoride. The mullitization temperature of the precipitates was 900 °C, and they grew into mullite whiskers at 1100 °C. The whiskers grew preferentially along the direction parallel to the c-axis, resulting in an orthorhombic-type crystallographic structure. XRD and FE-SEM results show that the whiskers were in high purity with a high yield. The whiskers had the aspect ratio of >15 (100 nm in diameter). The growth process of mullite whiskers wasdominated by a vapor–solid (VS) mechanism combined with the inducement of =Al–O–Si≡ bonds formed in the precipitates were beneficial for the low mullitization temperature and whiskers growth. The AlF_3_ amount was optimized to be 1.3 of the theoretical amount, which ensured appropriate AlF_3_ vapor supersaturation concentration. With the help of the vapor promoted mass transport process, precipitates particles eventually grew into mullite whiskers.
